# Initial stakeholder perceptions around opioid settlement funds in Pennsylvania

**DOI:** 10.1186/s13011-025-00665-7

**Published:** 2025-10-16

**Authors:** Halie Kampman, Kristina Brant, Maya Weinberg, Glenn Sterner, Brian King

**Affiliations:** 1 Department of Geography, The Pennsylvania State University, 302 Walker Building, University Park, PA 16802 USA; 2 Department of Agricultural Economics, Sociology, and Education, The Pennsylvania State University, University Park, PA 16802 USA; 3 Criminal Justice Research Center, The Pennsylvania State University, 1600 Woodland Road, Abington, PA 19001 USA

**Keywords:** Opioids, Epidemic, Pennsylvania, Opioid settlement, Decision-making

## Abstract

**Background:**

The opioid epidemic continues to pose significant challenges in the United States, with over 75% of nearly 107,000 drug overdose deaths in 2022 involving opioids. Recently, efforts to curb the epidemic have made strides. Beginning in the early 2000s, local and state governments filed a series of lawsuits against opioid manufacturers and distributors. The lawsuits held that manufacturers and distributors knew about the potential addictive nature of these painkillers and withheld this information from providers, leading to overprescription and overdose. The current settlement funds allotted to Pennsylvania amount to approximately 2 billion USD, can be spent over the next 18 years, and are distributed at the county level. This paper investigates the early perceptions of local government officials and substance use service providers in Pennsylvania regarding the rollout of settlement funds and their decisions about how to use them.

**Methods:**

This paper draws on 66 semi-structured interviews and 6 focus groups conducted in 2023 across six Pennsylvania counties. Interviews were audio-recorded, transcribed and inductively coded using NVivo software.

**Results:**

Findings reveal an initial sense of confusion among participants about navigating the funding process, exacerbated by unclear guidelines and insufficient support from state-level officials. Despite these challenges, participants express optimism about the potential for innovative, locally focused projects to effectively address the impacts of the epidemic. Emphasizing the importance of community involvement and transparency, the study highlights a collective commitment to ensuring that funds reach those most affected by the crisis. Yet still, some participants recognized the limits of the settlement funds in addressing the deep trauma that their communities have experienced.

**Conclusions:**

The recommendations underscore the necessity for better systems to outline funding processes, promote best practices, and enhance inter-county collaboration. This research contributes valuable insights into the decision-making dynamics surrounding opioid settlement funds and lays the groundwork for future investigations into their long-term impacts and efficacy.

## Background

The opioid epidemic that began in the late 1990 s in the United States remains an ongoing public health crisis. Since 1999, the number of opioid-related deaths has been rising continuously [3]. Of the nearly 107,000 drug overdose deaths in 2022, over seventy-five percent of them involved an opioid (ibid.). While the opioid crisis is far from being resolved, efforts to curb this epidemic have recently made important strides. Beginning in the early 2000 s, local and state governments filed a series of lawsuits against opioid manufacturers and distributors for fueling the epidemic by funneling huge amounts of prescription opioid painkillers (e.g., OxyContin, a brand of oxycodone) into communities [9]. These lawsuits hold that manufacturers and distributors knew about the potential addictive nature of these painkillers and withheld this information from providers, leading to overprescription and a proliferation of opioid use disorders [12]. These initial lawsuits coalesced into a national conglomeration of unified states and territorial claims known as a multidistrict litigation lawsuit in 2017 [17]. Further litigation is ongoing with additional companies complicit in the distribution of prescription opioid painkillers in the U.S., with a current total of $55.2 billion, anticipated to increase in the coming years [6]. These pharmaceutical companies are already distributing these funds back to the states, tribal communities, and territories to compensate for their role in the harms caused by the epidemic, and they will continue to do so for at least the next 18 years, according to the terms of the settlement [13]. Yet with this new channel of funds come myriad questions about how and where they should be spent.

There is evidence to suggest that under certain conditions, stakeholder engagement in health policy and management can be key to tailoring broad-based policy to specific populations [5, 11]. We are interested in the phenomenon of perceptions as a lens for understanding the underlying values, beliefs, attitudes, and interests predicating early-stage funding decisions. Since the settlement was announced, a growing body of literature in public health and medicine has focused on disseminating evidence-based recommendations for the settlement funds [15, 18] (Alexander and Mansour 2022[1]). Recommendations to guide effective expenditures are based in part on avoiding mistakes of the past, particularly in the wake of the tobacco settlements in 1998, which largely failed to reach the communities most affected [8]. Short- and medium-term recommendations for how to spend opioid abatement funds focus on investment in expanded harm reduction services that offer medication assisted treatment, naloxone, and social programs that serve people that use drugs [8, 13]. Long-term recommendations address the structural causes of drug misuse, calling for investment in programs that reduce homelessness, and diversion away from the criminal legal system and towards health services [8]. Notably, there is no national-level requirement to measure the impact of activities on the abatement of the opioid epidemic, which could result in less efficacious approaches [13]. Individually, states may elect to implement monitoring and evaluation plans. This has led some analysts to suggest that state and local expenditures should be overseen by federal entities such as the Department of Health and Human Services [7].

Despite the lack of a national settlement requirement for states and localities to report funds’ uses and evaluate their impact, researchers and practitioners have begun to investigate the ways that funds are being spent (e.g. appalachiaopioidremediation.org). Independent evaluations of funding allocation to date reveal instances of spending on prevention and harm reduction largely among liberal enclaves, but in equal part spending on law enforcement and prisons [15]. There remains a continued need for transparency: opioidsettlementtracker.com determined that only 21 states so far promise to publicly report 100 percent of their remediations. In addition to the ongoing need for evaluations and transparency, it will also be important to understand how the local entities tasked with spending the funds perceive of the funding structure, what they intend to do with the funding, and how they envision the particular needs and aims of their locality. To date, these topics have been underreported in the academic literature, which reduces the potential to identify best practices for public health interventions.

Using the case of the Commonwealth of Pennsylvania, this paper explores how local decision makers and other community members perceive the settlement funds in the early stages of their distribution. The current settlement funds allotted to Pennsylvania amount to approximately $2 billion (opioidsettlementtracker.com), this amount likely will change over time as further lawsuits are settled. Funds began to be distributed in 2022 with seventy percent going to county governments in Pennsylvania, fifteen percent to the Commonwealth legislature (who distribute the funding as part of their annual budgeting process), and fifteen percent to the litigating subdivisions (i.e., individual entities that leveraged lawsuits before the multidistrict litigation formation including county, city, and township governments; and District Attorney’s offices) [13]. Funds management is in the hands of The Pennsylvania Opioid Misuse and Addiction Abatement Trust (the “Trust”), which was established in May of 2022 and is governed by a Board of Trustees, made up of 13 members. These members are appointed by various entities across the Commonwealth, negotiated in the litigation process, including the Governor’s Office, the Pennsylvania Legislature, and county stakeholders (e.g., commissioners, substance use treatment administrators). This is an independent, non-profit Trust that is outside of state government, and while trustees may be associated with state governmental agencies, the Trust has no role in coordinating extant substance use intervention or funding deployment with the Commonwealth, these decisions are left to local and state administrators. The Trust is tasked with disbursing the funds on a yearly basis, ensuring that they are used for opioid abatement, and spent within the 18-month timeline outlined in the Pennsylvania settlement.

Guidelines for using the funds are outlined in “Exhibit E”, a detailed list of approved activities for opioid abatement (Nationalopioidsettlement.com). General categories include activities in opioid misuse prevention, harm reduction, treatment, and recovery services. The Trust provides no guidance or prioritization to county and state governments in Pennsylvania in their allocation of funding, except to ensure they are in compliance with the outlined accepted uses in Exhibit E. As of March 1, 2024, the Trust reported that Pennsylvania counties and subdivisions have reported spending or committing to more than $70 million (paopioidtrust.org). This amounts to more than two thirds of the total funds disbursed in that time period ($102 million) (ibid). This spending represents the work of more than 380 programs reported so far (ibid). Reporting on spending at the county level varies between counties. 

Allegheny county (where Pittsburgh is located) publicly shared detailed reports on expenditures to date. As of August 2025, they reported over $16 million (48% of total budget). Of their budgeted funds, they plan to commit 80% to treatment, 18% to prevention (and 2% to other causes) [2].

In Pennsylvania, since 85 percent of funds have been devolved to counties and subdivisions, decisions on how to spend the state’s allotted funds are largely in the hands of county-level leaders; these leaders have the power to choose what activities to prioritize and how to implement them. This structure presents a notable opportunity to reach the very communities that were, and often continue to be, harmed by the opioid epidemic. This paper addresses this opportunity by asking: What are county-level key informants’ initial perspectives on the process of receiving and spending newly available opioid settlement funds in their county? This paper is part of a broader project that aims to conduct a longitudinal evaluation of the impact of diverse abatement activities and interventions implemented across the Commonwealth on opioid-related outcomes.

## Methods

The study design is based in a constructivist paradigm, with the aim of understanding how key informants *perceive* settlement funds, rather than attempting to *predict* how they will be managed or spent. We draw on semi-structured focus groups and interviews conducted in 2023 across six Pennsylvania counties. These focus groups and interviews occurred very early in the distributions of funds, just after the first two lawsuit payments (September 2022, and December 2022).

The six counties were selected to include demographic and geographic diversity across the Commonwealth, to ensure that we captured perceptions on whether and how funds should be used to reach historically underserved groups and places. One county has a sizable Hispanic or Latino population (i.e., greater than the national proportion of 19.5 percent), and another has a sizable Black or African American population (i.e., greater than the national proportion of 13.7) [16]. Three of the counties are designated “rural” by the Center for Rural Pennsylvania [4], the state legislative agency focused on research and policy initiatives pertaining to rural areas. Two counties are in the Eastern region, two in the Central region, and two in the Western region. Finally, two of these counties are Appalachian, as designated by the Appalachian Regional Commission.

It is also important to note that the counties represent a range of harms experienced by the opioid overdose crisis. Across years 2015–2019, the peak annual overdose death rate for the county in our sample with the highest level of harm experienced was nearly three times as high as the peak annual overdose death rate for the county in our sample with the lowest level of harm experienced [10]. It is important to include this range, as counties in Pennsylvania receive allotments of settlement funds that correspond to the harm incurred. In other words, counties experiencing more deaths ultimately receive larger allotments of funds than those experiencing fewer deaths [13].

County-level leaders from these six counties were initially contacted through email to invite counties to participate in the study. In this initial outreach, county leaders were asked to first assemble a focus group consisting of key informants who were closely involved in the settlement decision-making process in their county. These county-level leaders who were initially contacted differed by county. In counties where the study team knew who was leading the settlement efforts, the study team reached out directly to those individuals and asked them to assemble a focus group, which would include those individuals themselves. In counties where the study team did not know who was leading the settlement efforts, the study team reached out to county commissioners (i.e., the lead public officials in the county) and asked county commissioners to assemble a focus group, which may or may not include the commissioners themselves.

Study team members reached out to this group of suggested focus group participants to request participation in a focus group discussion and set a time. Ultimately, one in-person focus group was convened in each county, composed of two to three participants. The purpose of focus groups was to establish contact with the participating counties, ensure that counties were interested in participating, and get a sense of the county’s specific processes and structures for the settlement rollout. Toward this end, focus groups followed a semi-structured discussion guide largely focused on three areas: participants’ backgrounds and involvement in the opioid settlement, their county’s existing data collection processes on opioid abatement efforts, and their larger interest in engaging in opioid settlement-related evaluation and assistance activities. However, an additional purpose of the focus groups was to inform the subsequent interviews. Focus group participants were given the interview guide and asked to provide feedback on additional areas that we should inquire about in interviews. Thus, many focus group discussions also ended up including conversations on answers to the questions in the interview guide. All focus group discussions were audio-recorded and lasted between one and two hours.

After focus groups were completed, the focus group participants were asked to recommend a list of key informants to participate in interviews. It was requested that these suggested key informants either participate in county-level distribution of funds or work in fields related to substance use services. Focus group participants were also invited to participate in interviews themselves. These suggested key informants and interested focus group participants were contacted through email or by phone. Interviews were either in person or via Zoom, depending on the participant’s preference. The purpose of the interviews was to delve into perceptions of the rollout of the settlement at the state and county level. Thus, the interviews used a semi-structured interview guide focused on four general topics: participants’ perceptions about the substance use landscape in their county, their knowledge about the settlement in general and the local process to use the settlement funds in their county, their perspectives on how the settlement funds are currently being used, and their ideas for how funds *should* be used. Interviewers also asked follow-up questions for clarification and elaboration. All interviews were audio-recorded and lasted between thirty minutes and one hour. Both focus group and interview recordings were transcribed using rev.com.

Table 1 details the research participants. Across focus groups and interviews, participants included substance use services professionals (i.e., prevention specialist at a recovery center, transitional housing director, emergency room physician), non-elected local government employees (i.e. county level human services, prison staff, police), elected officials (i.e. county commissioners), and local drug and alcohol department staff (i.e. a government agency, a non-profit agency, or a private company contracted to serve as the county drug and alcohol department). We did not specifically recruit people with personal lived experience, but this category was represented by a few individuals working in the substance use services sector. Three participants did not fall into any of these categories and were classified as other (e.g., a local professor). All focus group and interview participants provided informed consent and were offered a $50 Visa gift card for their participation. The study was approved by the Pennsylvania State University Institutional Review Board.

**Table 1:** Participants

Category	Focus groups participants	Key informant participants
Substance use services professional	3	25
Non-elected local government employee	4	15
Elected official	1	13
Local drug and alcohol department staff	7	11
Other	0	2
Total	15	66

Transcripts from focus groups and interviews were inductively coded by three of this manuscript’s authors [14]. First, the three team members each reviewed the transcripts to inductively generate an extensive list of themes. These were then collectively condensed into a preliminary list of codes that speak to primary participant perceptions. These codes were tested out on three interviews, with each of the team members coding independently. Team members came together to compare and ensure consistency in coding. When discrepancies arose around a code the team collectively revised its definition for clarity. Once the codes were finalized, all transcripts were uploaded into NVivo software where they were divided among the three team members and coded independently. The team met weekly to review coded transcripts, discuss questions that emerged, and maintain consistency. Few discrepancies arose, pointing to general consistency in data interpretation. The subsections of the results section of this manuscript are structured around the primary perception codes, reflecting a comprehensive picture of participant perspectives.

## Results

Interviews exposed a diversity of perspectives about the settlement funds. Participants often felt an overwhelming sense of confusion about how to manage and spend these new funds. Yet also, they recognized the opportunity for innovation in strategies to opioid abatement, with more bandwidth to explore a variety of approaches. Consistent among the participants was the importance of going local, in the sense of investing in community-based projects and programs. It was important to participants to make the funds count and maintain transparency in their operations. Finally, to many participants, there were clear limits to these funds, as well as an overall recognition of the collective trauma of the opioid epidemic across their communities.

### An overwhelming sense of confusion

Across the different counties included in this study, the most common perception was a sense of overwhelming confusion regarding the details of the settlement. This confusion was felt among different types of participants—including those intimately involved with settlement decision-making in their counties, those who work in local substance use organizations, and the general public.

For those tasked with making decisions about the funds—either county commissioners or the entities to whom they had passed decision-making power—this confusion concerned how to spend settlement funds. These participants often had basic knowledge about the purpose of the settlement, the general structure of the settlement, and where to find rules about how to spend settlement dollars (i.e., “Exhibit E”). However, many explained that they lacked more detailed information they needed to make decisions for their counties—such as what specific activities could be funded or not and what information they needed to collect for reporting purposes. One local elected official told us:

“There’s been a whole lot of stuff that we don’t know—more than we do know. And now we’re running with bags of money through the community and [we’re] not sure how we can spend it, or if we can spend it.” (FG2)

What intensified this confusion was an uncertain yet urgent timeline. Participants knew that county allocations needed to be spent on an annual basis, and subsequent years’ funds would not be dispensed if a county did not spend their previous year’s balance. This understanding created urgency: yet simultaneously, these decision-makers did not necessarily know the exact dates of when funds would be dispensed, when they needed to be spent by, or if they needed to be committed or actually disbursed. One local government worker recalled:

“Originally when this concept was brought to us, [our] Commissioner was kind of in a panic mode… We were basically put in panic mode to come up with some ideas on how we're going to spend this, because the first installment of funds was going to be rather substantial. And we have a timeframe of being able to use that.” (KI57)

What’s more, several participants told us that there is little assistance available to counties in making these decisions, and they did not know where to go to ask questions for either advice or clarifications. They looked to the Trust for instructions and answers but did not feel like the Trust was providing what they needed. The elected official quoted earlier continued:

“We don’t want to put our county in a bind where we spend $800,000 or $2 million and then someone comes down five years from now and says, ‘You could’ve spent it that way.’ That's why we’d like to know up front: is this okay to use it in this way? There just seems to be no resource there, no easy resource to tap into that… There hasn’t been, from my opinion, a lot of great information coming down.” (FG2)

Because county-level decision-makers were confused about settlement rules and processes themselves, many could not provide information or answers to the substance use service providers in their communities who had questions. Thus, this sense of confusion filtered down from decision-makers to others. Those service providers whom we interviewed tended to know about the settlement in general, but most were unclear about what was going on in their specific counties. Some knew who was overseeing, or would be overseeing, funds in their counties, but they still could not find out details of how the settlement would be distributed. One professional in the substance use services sector relayed that he would ask his county decision-makers for details but could not get answers:

“We were hearing about [the settlement] out there. We knew it was coming. And we were asking,"What's happening? What's happening with this money?"I know it would get asked a lot at council meetings and things like that. The answer was always… ‘It’s in the works, it’s in the works, we can’t say anything right now.’” (KI27)

In other interviews though, we met service providers who were not even aware of who in their county held this control—in other words, whom to even ask to find out what was going on.

Finally, while our participants—as people engaged in substance use-related work—tended to know about the settlement in general, even if they lacked details about what was going on in their counties, they expressed that community members generally may not even be aware of the settlement’s existence or realize that so much of the funds would be coming to individual counties. One local government worker told us:

“I would say if we interviewed a hundred people on the street, I could tell you zero of a hundred know that there’s an opioid settlement. Which is unfortunate, because they probably know people. Either they have family, loved ones, or even themselves that don’t know that this money’s available to be put to good use.” (KI22)

Some county decision-makers told us that they were attempting to share information with the community but did not know how to best reach them. Others were holding off on sharing anything until they had gotten more answers themselves.

While confusion was widespread, there was one notable exception to this theme. The Trust itself is made up of individuals who live across Pennsylvania. Thus, some counties have residents who are members of the Trust. This was the case in one of the counties included in our sample. Confusion was lessened in this county due to the direct line of communication between the county and the Trust. Certain key stakeholders in this county seemed to have less anxiety around the rollout of the settlement due to having a close connection with a source of information and knowledge.

### Excitement about potential innovation

Despite this overall sense of confusion felt across counties, participants recognized that flexibility was a key element of the settlement’s guidelines. While funded activities needed to fall within the scope of Exhibit E’s outline, this outline was rather comprehensive of most project types within prevention, treatment, recovery, and harm reduction. They saw this flexibility as an opportunity to both fulfill “wishlists” of existing ideas and to try out new and creative approaches to addressing the epidemic in their community (KI15). One employee of a county drug and alcohol department spoke about the potential she sees for this type of unrestricted funding:

“I hope that it's going to bring lasting change to our community. I hope that we can build a community that is so ready, so recovery ready, that anybody that wants treatment, seeking treatment, knows where to get it, knows that we have innovative ways to serve them, and that we're open to multiple pathways.” (KI62)

This participant highlighted that because the funding could be used for multiple and diverse activities, counties have the potential to fund different projects that work together to build multifaceted and multisector community solutions.

Because of this flexibility in guidelines, and because counties can change their funding priorities every year, some participants acknowledged that they also could try different innovations to see what works. A drug and alcohol department employee from a different county shared that she and her team are “willing to try anything, really, within the bounds. And if it doesn’t work, we can back off. But I feel like you don't know until you try it. We have the ability to do that” (KI39). Counties would be able to continue funding successful innovations or step away from those that did not prove worthy of future investment.

Relatedly, the flexibility of Exhibit E was recognized by participants as an opportunity to fund projects and organizations at smaller scales which larger, more targeted grants often overlook. This feature was understood as particularly meaningful because, often times, those organizations are working most directly with affected populations. A participant working for their county’s drug and alcohol department shared:

“What we found is, especially as you’re doing some of these smaller organizations, they can't deal with a contract with all these requirements and audits and this and that, this and that. There's some room for some, I don’t want to say play, but for being flexible within.” (FG2)

Funding was seen as a unique opportunity to realize smaller projects, free from a hefty set of requirements like lengthy application processes, complex reporting guidelines, and strict budgetary rules.

While there was clear enthusiasm across most participants for the opportunity to lean into the creativity that the funding model allows, many shared fears about how some decision-makers might ultimately choose to invest in existing initiatives instead. One elected official expressed concern for the “inherent bias” that would lead decision-makers to use funds “just to allow us to keep doing what we are doing” rather than “[opening] it up a little bit” (KI42). Several participants also noted that because few organizations have had such flexibility in the past for how to spend their funding, it may be difficult to come up with novel solutions. A local government worker reflected, “I think for this first round, I think what we saw was very few entities kind of thinking outside the box, because they've never had the opportunity to do that, right?” (KI61).

Finally, participants acknowledged that there are limitations to the settlement dollars’ flexibility that can occasionally get in the way of implementing some possible solutions. In particular, participants believed that restrictions on how much can be spent on administrative needs, and the inability to use funds to support initiatives that address underlying causes of opioid use, could act as barriers to creative solutions (KI47).

### Importance of funding local

Building on excitement about the flexibility of settlement funding, participants were also enthusiastic about the local decision-making autonomy possible through devolution of dollars to the county level. Participants believed that funding at the “ground level” allows money to be funneled to what is already known to work in local contexts (KI22). The idea that “local problems deserve local solutions” was echoed across participants, highlighting the adaptability of the funding model to reflect specific community needs (KI15). One local government worker also emphasized that local control could allow for funds to respond more rapidly to emerging issues and lessons:

“Once we get moving and these programs are launched and we're able to get some data on the outcomes and success of certain programs, really tailoring it to be the most effective use of funds...knowing that any plan will be revised depending upon what's going on in the community, how well certain initiatives are taking off, if we need to adjust”. (FG3)

Participants acknowledged that when funding allocations and investments can be adapted and “escalated” in real-time, plans and investments can be dynamic and reflective of both existing and emerging realities (KI36).

While initially, the confusion among county-level decision makers about what funds could be spent on led to "a slow start” in fund disbursal, many believed that in the long run, local allocation would be “much more efficient” when free of the bureaucracy of state and federal control of the dollars (FG3; FG22). Fresh on the minds of several participants were memories of how little tobacco settlement funds filtered down to local entities. A professional in the substance use services sector shared:

“[There was] a lot of bitterness about how that went. That basically as the money was working through the pipeline for the settlement, it got completely absorbed by large entities and large state programs and it didn’t really make it down to the grassroots level and therefore it didn't have the impact that was desired. And so people have really paid attention to that process in our state and saying it needs to get through. That cost is being borne by the county to a large extent.” (KI8)

Some participants believed that the decision to devolve such a large percentage of funds to the counties may have even reflected policymakers’ awareness of widespread dissatisfaction with the tobacco settlement funding model. They expressed appreciation that the opioid settlements funds will instead gather knowledge “directly” from local experts and “really get the money into the community they serve” (KI 19).

Several participants also remarked how the funding model allows for local decision-makers to listen to a diversity of community voices to determine how funds are spent. A localized process could allow community members to get involved, speaking to their local county officials or attending community forums where ideas could be discussed. A professional in the substance use services sector emphasized:

**“**The guidelines are so varied that it gives those local communities opportunities to look at the menu and find out from community members, ‘How can we help resolve this problem together?’ It’s a collective. It’s a collaborative that really helps the community as a whole get well as a whole. I am a real believer in it takes a village. It really does take a village to resolve this. All hands on deck at this point. All hands on deck.” (KI15)

Participants such as this individual believed bringing in new and different stakeholders to the funding process could unite the community in new ways.

Finally, while generally eager and enthusiastic about the potential impacts of local funding, participants wanted to know what other counties are doing to expand on and adapt best practices to their own context. A local drug and alcohol official requested “resources for counties to see what other counties are doing and how it's helping them. I think kind of a general resource like that would be helpful for everybody.” (KI 9).

### Importance of making the funds count

Among participants, there was a recognition of the weight of the responsibility that comes with the opportunity to allocate settlement funds. This recognition manifested in a commitment to “make the funds count,” or to use the funds wisely to make the greatest impact in their communities. This commitment was articulated as an intimate obligation, a commitment to public service, and a need to prevent parallel epidemics in the future.

Participants’ sense of obligation to use the funds wisely was often laden with emotion. They recognized that these funds were designed in part to right the wrongs of the opioid crisis and to address the tragedy of opioid misuse in their communities. An elected official explained how “as leaders, that's the only hope we can provide families that have lost loved ones to this crisis. I mean, I feel a real obligation” (KI36). The counties’ role was often framed in intimate terms. Participants would tell stories about how they lost friends or colleagues to the opioid epidemic, motivating their personal and professional commitment to future abatement.

Participants also pointed to a need to ensure that the public sector was doing its due diligence to make the funds go as far as possible. There was often a feeling that decision-makers’ actions were being scrutinized, fueling a sense of duty to do the right thing. A professional in substance use services explained that “we don't want to come out with egg on our face. We don’t want to come out with 'Pennsylvania wasted its money, or [this] county wasted its money.'” (KI59). Some participants were preoccupied with structural limitations of this pool of funds: particularly when funneled through the public sector, they recognized the risk of funds being misdirected or wasted. A professional in substance use services explained how challenging it was to find enough resources to sustainably manage the funds: “so there needs to be an entity that’s not short-staffed and shortsighted and easily duped that keeps a strong eye on these endeavors and the recipients of these funds.” (KI18). Participants agreed on the need to commit adequate resources to funds management, yet it was difficult to find the funds to support this, as staff in the substance use treatment area are already stretched thin.

Participants had an eye on the future, making sure that the funds still counted even after they ran out. A professional in the substance use services sector asked, “Are we just trying to make a splash, or are we really trying to help the community push it forward permanently towards sustainable, and able to move forward without us being here?” (KI49). Others shared concerns about the timeline: while eighteen years is long, it requires a consistent vision to follow through with the expenditures.

### Concerns about transparency

A specific commitment to transparency ran parallel with the general impulse to make funds count. The commitment to transparency was articulated in discussions of how to design measurable outcomes, and in conversations about who is invited to the table in making decisions.

Because the settlement funds were newly disbursed, participants were in the process of developing their plans to monitor outcomes. It was important to many that these be publicly shared and concrete. One local government worker spoke to ways that her county decision-makers were pursuing transparency: “For me, I am excited for our website to come out and then some of this funding to start to flow through various contracts and then be able to show, ‘This is what we’ve done. We purchased the Narcan, there's Fentanyl test strips’, but then those are […] concrete.” (KI 40) Some expressed hesitation about their ability to create and enact a reliable structure. With the 18-year timeline for fund distribution there was concern about the need to establish a system and a precedent. A staff member at a local drug and alcohol department spoke to the challenges of maintaining transparency:

“We as prevention providers are really, really into figuring out better ways to impact or to capture the impact that we made…We actually want to do a better job, but…We’re not data statistic experts. We have to probably buy a program or have someone come in and train us, or whatever. That would be helpful.” (KI4)

Others mentioned the need for “toolkits” or “databases” or a desire to hire external evaluation specialists.

It was also important to participants to invite the community into decision-making processes in order to maintain transparency. For instance, community forums were considered to be a way to inform local people about how funds were being spent, and to elicit their opinions. Yet still there was a concern that this type of community engagement was aspirational but would be difficult to effectively carry out. A professional in the substance use service sector had insight into who could be expected to be engaged: “I feel like now do you go to commissioner’s meetings if you're a regular Joe […]? I don’t know.” (K11). Other participants pinpointed specific populations they did not think were aware of the funding opportunities or decisions. For example, one local government worker added:

“The city is literally about 70% Latino. How do we get out into those communities and those neighborhoods? We have to go to where they are. That’s another phase. We have to go to where they are and explain this to them, because they're not reading the [local newspaper]. They're not going to read the magazine.” (KI22)

These concerns called into question whether the communities that the funds intend to serve are adequately included in decision-making processes.

### Recognition of community trauma and the settlement's limits

As the above sections have demonstrated, participants certainly believed that the settlement funds could make important changes in their communities, and many were committed to figuring out how to maximize the funds’ impact. While there was considerable confusion and some related anxiety about the process of using funds, there was an air of excitement about what programs and results could come from this money. Nonetheless, some participants had a more resigned and melancholy tone; these participants recognized the deep trauma their communities had experienced and pointed to the limits of settlement funds to address that trauma.

Some participants pointed to the inability of the funds to turn back harms already created. One professional in the substance use services sector told us:

“My one hope is that we don’t forget why this money is here. This money is coming from this greed, and lies, and all of this stuff that just tarnished an entire generation of people… There’s not really a monetary value that you can put on these things… I love to see Big Pharma pay out. But, it’s not going to bring back the dead… I’m glad that this money’s available, but ultimately for me, as professional and personal, it's a little too late. You know? All my friends are already dead.”

This participant, alongside several others, noted that it is impossible “to put a value on a life” (KI 12). While they appreciated that pharmaceutical companies were being held accountable, no amount of money paid out could undo the deaths that came from their actions.

Others noted the limits of the funds to assist those who had been most impacted. In one county’s focus group, participants emphasized how they had been seeking input from the public to decide how to use the funds. Yet, they also noted that there were suggestions they could not follow. Specifically, one of the focus group participants, a local drug and alcohol department staff member reported that some grandparents raising grandchildren believed that funds should be directed to them to support raising children whose parents had died:

Even as recently as last week, I sat in a room with a parent [of an overdose victim]—a grandparent [raising their grandchildren]—who said, these should be our funds. And so, it was a miss, they think, for the Attorney General’s office—or whomever decided how the funds would be expended—to not provide remediation, reparation, whatever term for their suffering. (FG3)

This participant noted that funds had guidelines, as established by “Exhibit E.” They could not give a payout to every family who experienced an overdose death, even if that is how some of the public believes funds would be most fairly spent.

Recognition of community trauma and the limits of the settlement to reverse the harm caused by the overdose crisis was not confined to a particular type of stakeholder. However, these sentiments were most often expressed by participants who had a direct connection to harm—either through their own lived experience or through the lived experience of a loved one. Those who had direct connections to harm were distributed across participant type. For example, some elected officials referenced family members’ experiences, while several substance use services professionals reflected on their own journeys with addiction and recovery. Direct experiences seemed to heighten the understanding of the vast harm caused by the overdose crisis and the settlement’s inability to fully recoup these losses.

## Discussion

The participant perspectives examined in this paper contribute to a growing base of knowledge about what is happening with the opioid settlement on the ground. Early in the process, most participants felt an overwhelming sense of confusion when trying to navigate the new funds. While funds and spending timelines were set, specific processes for how to disperse them remained obscured by sets of rules (i.e. “Exhibit E”) and the perceived lack of detailed information in Pennsylvania. The lack of assistance from the Trust, the state entity overseeing Pennsylvania’s settlement allocation, was also a key concern for participants. Despite this confusion, there remained substantial excitement about the potential for these funds to advance innovative projects. The funds allowed a certain amount of freedom, which participants believed could allow them to try out new and innovative approaches to abatement and see what works. This flexibility was important to participants, though they acknowledged that there were still some rules that hindered their ability to implement “root cause” solutions outside the bounds of Exhibit E. A value that was common to participants was the importance of funding local projects, perceived to be much more efficient and unconfined from state or federal bureaucracy. Looking back on the failures of past settlements, like the tobacco settlement in the 1990 s, participants were committed to putting funds towards the communities who suffered most from the epidemic. Participants credit local approaches with helping to make the funds count, borne from a recognition of the weight of the responsibility of their role in abating a deeply personal and intimate issue. There was a collective feeling that the public sector needed to do right by the community members, with an eye on future sustainability. To do so, the maintenance of transparency was key, and participants were interested in seeking transparency through data collection, external review, and evaluation. Participants also believed community involvement could help to maintain a high bar of transparency. Finally, the participants’ actions were shaped by the universal recognition of the deep trauma that their communities had experienced, but with a sense of concern about the ultimately limited ability of the settlement funds to address this trauma.

While existing scholarship tends to focus on agenda setting, without considering *how* the agendas are set, this paper is situated in a unique way. Our findings can help to inform the actual decision-making processes by which funds are allocated, with a particular sensitivity to contextual constraints and concerns. Adding to existing scholarship that makes recommendations about how money can be spent [15, 18], (Alexander and Mansour 2022), and bolstering work on the potential of stakeholder engagement for tailoring contextual policy change [5, 11], our data sets us up to make recommendations about how states can help counties in the process of spending funds.

Participants’ perspectives can be translated into general recommendations that could be used to improve funds oversight and spending. First, based on articulations of confusion and feeling overwhelmed, there is a clear need for better systems of outlining details about settlement distribution process, answering questions, and even providing technical assistance. This can benefit decision makers by reducing confusion, reticence for implementing interventions funded by the settlement, and paving the way for innovative and exploratory projects to develop. Additionally, participants’ desire to fund local projects that “make the funds count” is supported by the breadth of the guidelines outlined in Exhibit E and should be maintained by ongoing county-level commitments. Funding local could be further enhanced through a particular focus on harm reduction programs, which other studies have recommended in the short and long term [8, 13]. Other scholars have also called for transparency in spending funds, particular in the wake of the mistakes made in the tobacco settlement [7, 13, 15]. As others have argued, concern about the need for transparency and the primacy of making the funds count can be addressed by a more concerted effort to identify best practices and evidence-based practices informed by local public health data. This would allow for counties to learn about what one another is doing, supporting counties in evaluating their decisions. Greater communication and transparency could also be advanced through independent research consortiums (such as the one that this manuscript’s authors are a part of), which collect and publicly share data on funds distribution (paopioidsettlementdata.org). Such resources allow counties to read detailed reports of one another’s projects.

## Conclusion

This paper provides valuable insight into the innerworkings of settlement decision making, paving the way for future research. It remains to be seen to what degree the experience of participants in Pennsylvania may be translatable to other states. Future research that examines whether the participants of this study are upholding the principles that they spoke about can help to reinforce, or to challenge, the themes that emerged in this early-stage data set. For instance, it would be interesting to explore the degree to which funding local, innovative projects is pursued along the course of settlement spending, or whether transparency is maintained through community involvement. It is important to note that developments have occurred since data for this paper was initially connected, and given that settlement funds can be redirected annually, there is a need for continuous evaluation of their impacts.

This approach is potentially limited in several ways. Due to the time-intensive demands of semi-structured in-person interviews, only six of Pennsylvania’s 67 counties were reached. Though saturation was reached for these included counties at the completion of data collection, it is possible that alternative perspectives from other counties are not represented in this study. At the county level, perceptions are mostly limited to those of people poised to make decisions about the settlement funds, rather than with general community members. While the general community is not directly represented by our sample, several decision makers referenced perceptions that they heard from community members, and some of the informants who worked in the substance use sector were also people in recovery from opioid use. While professionals were the best suited to speak to the focus of this paper – on *processes* of spending settlement funds – future research projects would benefit from learning from the perspectives of community members. It would be useful to understand more about what this money means to the people who have suffered from opioid use. The group of decision makers was broad (including elected officials, professionals in the substance abuse sector, government workers and healthcare professionals), but not comprehensive of all individuals involved in the settlement funds.

This study also only captures perceptions from a particular moment in time – the very early stages of fund distribution – and perceptions are likely to change and develop over time. These early data inform an essential baseline but should not be confused for comprehensive synopses of participant perceptions of settlement funds. Despite these limitations, this study fills a clear void in the literature on how local communities are perceiving and beginning to use opioid settlement funds. It provides an example for future research in other state contexts, while also laying the groundwork for longitudinal evaluations on localities’ uses of the funds.

## Data Availability

No datasets were generated or analysed during the current study.

## Appendix 1

### Focus group interview guide

Hi [*name*],thanks so much for taking the time to meet with me today. We really appreciate your willingness to develop this study together, so that we can gain the best information possible that will be helpful for you as well as the Elevate PA initiative.

I’d first like to review the consent information with you. Do you have the document that I sent you handy? Would you like me to read through this with you? [*Read through if respondent indicates that would be helpful. Have respondent sign consent form.*]

Before we begin, I want to remind you that your participation in this project is voluntary, and you are free to skip any questions you do not wish to answer. Your participation implies your voluntary consent to participate in the research. As a reminder, we will use the information collected across all of our interviews to develop the types of data and data collection methods for the Elevate PA initiative. The results will also inform a larger evaluation we are conducting on the impact of the opioid settlement funds on the landscape of opioid use disorder and overdose in Pennsylvania. Do you have any questions or concerns before we get started?

**Stakeholder background**

Okay great, let’s start with some introductory information about your background.

1. What is the title of your role within [*organization name*]?
2. Can you tell me what your key responsibilities are in that role?
3. How long have you served in that role?
4. How long have you been a part of [*organization name*] in general?
5. How long you have worked in [*county name*]?
  **Existing data reporting**
  Now I’d like to talk to you about the types of data that are already being collected and reported from [*county name*].
6. Can you tell me about the types of data and information on substance use related initiatives that are being collected here in [*county name*] and what it is being collected for?
  a How often is this data collected?
  b Who collects the data and who is it provided to?
  c How is the data collected?
  d Who analyzes the data?
  e Do you have any written reports that communicate out on this information?
    i. [*If yes*]: Could I have a copy?
    ii. [*If no*]: Do you plan to document this plan in the future? Could I have a copy when you do?
  f Are these data that you could potentially share with us?
7. Is there any data and information related to substance use that you wish you had access to?
8. Are there questions about substance use and substance use related initiatives that you would like answered that would be helpful to you?
  **Review of interview guide**
9. Now I’d like to review your suggestions for the interview guide I sent to you via email. Have you had a chance to review it?
  a [*If yes*]: Great! Thank you so much for reviewing it. Could you walk me through your suggestions?
  b [*If no*]: No problem. Let’s just go through the document together. [*allow participant the ability to talk through potential changes*]
10. Is there anything in this guide that you think is missing? Are there questions you would like answered that we could integrate into the guide?
11. Think of the people and organizations in [*county name*] who work in the substance use space or who are involved in the disbursal and use of settlement funds. Can you help us generate a list of whom to reach out to when doing these interviews?
  a [*After generating this list*]: Can you help us collect contact information for any of the people on this list?
    **Engagement strategy**
12. What additional data or information would be helpful to you as we collect data for the Elevate Pennsylvania initiative?
13. What type of data analyses, reports, and results would be helpful for you and [*county name*]?
14. How would you like to connect with others around the state to share information about what we’re learning in the Elevate Pennsylvania Initiative?

Before we finish up, is there anything else you think would be helpful for us to know as we start to collect this data?

Thanks again for being willing to share your time and work together on this initiative!

## Appendix 2

### Key stakeholder interview guide

Hi [*name*],thanks so much for taking the time to meet with me today. We really appreciate your willingness to share your knowledge of what is going on around substance use and the opioid settlement funds in [*county name*].

I’d first like to review the consent information with you. Do you have the document that I sent you handy? Would you like me to read through this with you? [*Read through if respondent indicates that would be helpful.*] 

Before we begin, I want to remind you that your participation in this project is voluntary, and you are free to skip any questions you do not wish to answer. Your participation implies your voluntary consent to participate in the research. As a reminder, we will use the information collected across all of our interviews to summarize the different ways that counties are deciding how to use their allotted funds from the settlement. The results will also inform a larger evaluation we are conducting on the impact of the opioid settlement funds on the landscape of opioid use disorder and overdose in Pennsylvania. Do you have any questions or concerns before we get started? 

**Stakeholder background **

Okay great, let’s start with some introductory information about your background and your own observations of [*county name*].

1. Can you tell me what your current role is in [*county name*] and what your key responsibilities are in that role?
2. Have you done any other related work in [*county name*]? What was that?
3. How long have you lived and/or worked in [*county name*]? 
4. Can you tell me how you’ve seen [*county name*] impacted by opioid use and overdose in the time you’ve lived or worked here? 
5. Can you tell me how the landscape of substance use and substance use services have changed in the time you’ve lived or worked here? 
6. What do you currently see as the most pressing issues in [*county name*] related to substance use?
  **Local structure and process for disbursing funds**
  Now I’d like to ask some questions about your knowledge about the opioid settlement funds and the process of disbursing funds here in [*county name*]. There are no right or wrong answers here.
7. First, can you tell me what you know about the settlement?
  1. [*If R needs help getting started]: *Do you know where the settlement funds are coming from, and why? Do you know what the funds are intended to accomplish? 
  2. [*If R does not know anything, provide a brief background about the settlement.* 
8. What do you know about the decision regarding how the settlement funds would be allocated across communities in Pennsylvania?
  1. *If R does not know anything, provide a brief explanation for how settlement funds were allocated across communities.*
  2. Do you think this system of allocation was fair? Why or why not? What do you think could have been a better system of allocation?
9. What do you know about who oversees the disbursal and monitoring of settlement funds in Pennsylvania?
  1. *If R does not know anything, provide a brief explanation of the trust.*
  2. Do you have any thoughts on whether or not this will be a fair and effective system of managing the funds? What do you think could have been a better system?
10. Do you think the general community in [*county name*] knows about the funding that will be available here?
  1. [*If yes]: *How was this communicated to the community? 
  2. [*If no]: *Do you think the community should be better informed and included? If so, how would this be done? 
11. Do you know who has been involved in discussing or deciding how the funds will be used here in *[county name*]*?*
12. Would you say that these people/groups typically work well together in other county activities, or no? Why is that?
13. Can you think of any people that it would make sense to include in this process who are not currently included?
  1. *[If yes]:* Who are they?
  2. *[If yes]: *Could you share why they are not included in the process?
14. Do you know if the people involved in disbursing the settlement funds in [*county name*]came up with a list of goals or priorities for the funds?
  1. [*If yes*]:How did they come up with this list of goals or priorities?
  2. [*If yes*]:What would you say are the biggest goals and priorities? 
  3. [*If no*]: Do you think this kind of planning should happen? How do you think it should go? 
15. Do you know if there is there a process by which interested organizations can request funding for specific activities? 
  1. *[If yes]: *How do organizations request funding? What do you think about this system? 
  2. *[If no]: *Do you think there should be a process for organizations to submit ideas and request funding? If so, what do you think that could look like? 
16. Do you feel that the county’s process of selecting activities to fund is transparent? Why or why not? 
17. Do you know if there is a plan in place for how funded activities will be evaluated?
  1. [*If yes]: *What is that plan? How will the activities be evaluated? 
  2. [*If no*]: Do you think there should be some kind of plan for evaluating the interventions? If so, what do you think it should look like? 
18. Other than what we have already discussed so far, do you have any other thoughts on the disbursal process in [*county name*]? Do youthink anything else should work differently in the process of how funds are disbursed in [*county name*]?
  **Local interventions** 
  I’d now like to turn to think about the specific activities that will be supported with the settlement funds. 
  *Note: If funds have not started being used yet, use language about what may be funded or is likely to be funded rather than what is already being funded.* 
19. Do you know if settlement funds are being used to support or bolster any substance use-related initiatives that were already in place before the settlement?
  1. [*If yes*]: Can you tell me what these initiatives are and who implements them or oversees them? 
  2. [*If yes*]: Do you think this is a good use of the funds? Why or why not? 
  3. [*If no]:* Do you have an idea of why funds aren’t being used to support any existing initiatives? Do you think they should be? 
20. Do you know if settlement funds are being used to support any new types of interventions that did not previously exist?
  1. [*If yes*]: Can you tell me what these new interventions are and who is implementing or overseeing them?
  2. [*If yes*]: Can you tell me what these new interventions are and who is implementing or overseeing them?
  3. [*If no*]: Do you have an idea of why funds aren’t being used to support any new types of initiatives? Do you think they should be?
21. Do you know if there are any specific kinds of activities that would probably not be funded here in [*county name*]? 
  1. [*If yes]*: What are those activities? 
  2. [*If yes*]: Why would they probably not be funded?
22. Thinking about the different types of interventions that currently exist or are currently being supported by the settlement funds, are there any specific interventions that you think are still needed in [*county name*]?
  1. [*If yes]: *What are they?
  2. [*If yes*]: Do you think the settlement funds could help these interventions come about in the future? Why or why not?
23. Thinking about the different types of populations that are currently being served by the interventions in *[county name*]*,* are there any specific groups that you think are still underserved?
  1. [*If yes:] *Who are they?
  2. [*If yes*]: Do you think the settlement funds could help better serve them in the future? Why or why not? 
24. What do you think are the strengths that [*county name*] has that will allow it to really utilize the settlement funds to its benefit?
25. What do you think are the challenges that [*county name*] will face in making the most of the settlement funds?

Before we finish up, is there anything else that you would like to share that could help us understand what is going on in [*county name*]?Is there anything that you think would be helpful to you or [*county name*] that we could assist with? 

Thank you so much for sharing your time and all of this information with us. 
